# Evaluation of Polyethylene-Based Long Lasting Treated Bed Net Netprotect on *Anopheles* Mosquitoes, Malaria Incidence, and Net Longivity in Western Kenya

**DOI:** 10.1155/2013/563957

**Published:** 2013-10-01

**Authors:** M. T. O. Odhiambo, O. Skovmand, J. M. Vulule, E. D. Kokwaro

**Affiliations:** ^1^Kenya Medical Research Institute, P.O. Box 1578, Kisumu, Kenya; ^2^Kenyatta University, P.O. Box 43844, Nairobi, Kenya; ^3^Intelligent Insect Control, 118 Chemin des Alouettes, Castelnau-le-Lez, France

## Abstract

We studied the effect on malaria incidence, mosquito abundance, net efficacy, net use rate, chemical analysis, and holes of a long lasting insecticide treated bed net (Netprotect) in western Kenya, 2007–2010. Nets were hung in 150 households 6 months before they were hung in a second, 2 km away. Indoor resting densities were monitored by pyrethrum spray catch and malaria cases by passive detection using clinical manifestations and rapid diagnostic test. The probability of finding *An. arabiensis* in the control area was 2.6 times higher than that in intervention area during the first 6 months. Human blood feeding index of *Anopheles funestus* declined 17%. After bed nets were hung in the second area, malaria incidence declined 25% down to the level in the first area. Incidence remained at this low level for 2 years. 90% of collected nets were efficacious after 3-year use. Deltamethrin dosage declined from 1.9 to 0.5 g/kg over 3 years. Attrition rate after 3 years was 21%. WHO hole index changed from 333 to 114 to 381 over the three years. This index summarizes the numbers of holes in size categories and multiplies with the mean hole area per category. It is very sensitive to the impact of big holes in a few nets.

## 1. Introduction

Large scale trials with insecticide treated bed nets (ITNs) have been shown to have a profound impact on reducing malaria transmission in experimental trials in sub-Saharan Africa and were recommended for large scale operations [1]. However, this tool did not become practical before the first long lasting insecticide nets (LLINs) were marketed and recommended by world health organization [2]. Preliminary WHO recommendations of LLIN are based on short term studies on vector impact and wash resistance. However, in sub-Saharan African settings, loss of insecticide is not primarily due to washing [3], but due to handling and evaporation. Dabire indicated that, though LLINs showed good efficacy on mosquitoes under controlled conditions, their effectiveness in the field conditions with respect to actual duration of insecticide protection in the field did not last for five years as indicated on the bed nets [2]. Recent research has shown that wear and tear may be more important for bed net durability than wash removal of insecticide [4]. This has led to the development of several hole indexes, and WHO recently modified the model it recommend. Since holes can only be measured on nets still present, it is the most meaningful when attrition rate can be followed, which means that cohorts of nets must be followed. However, the effect of gradually declining insecticide in the net, increasing the number of holes, and net disappearing has not been combined with observations of malaria incidence in field studies, and the effect is thus uncertain. This study was set up to see how an LLIN with a preliminary WHO recommendation performs in the field as a vector control tool, combining field observations of mosquito density to net parameters as insecticide load, bioassay performance, and hole index, and to see how the expected decline in efficacy impact malaria incidens. Netprotect bed nets were reportedly developed on the advantage of the first two long lasting bed nets using a fine mesh-like polyester net but having the strength and incorporation technology of a polyethylene nets. The bed net is made out of polyethylene mixture in which the insecticide (deltamethrin) is incorporated directly into the fabric at the rate of 1.8 g/kg or 60 mg/m^2^ [5].

## 2. Materials and Method

### 2.1. Study Area

**Figure 1:**
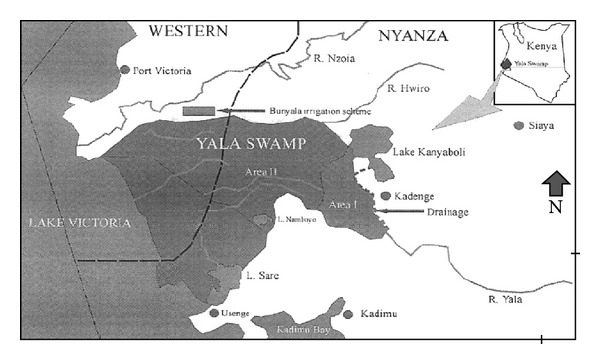
Map of Kanyaboli village [6].

Kanyaboli (Figure 1) is one of the villages clustered around the Dominion rice farm at the north shores of Victoria lake around the Yala swamp, about 70 km west of Kisumu town in Western Kenya. The area encompasses about 18.5 km^2^ and is adjacent to an oxbow lake of Kanyaboli. It has an estimated population of about 4000 people [7]. Yala swamp is one of the most important flood plain wetlands around Kanyaboli lake and one of the largest swamps in Kenya. The swamp forms the mouth of two rivers Yala and Nzoia and is a freshwater deltaic wetland arising from backflow of water from Victoria lake as well as the rivers flood water. The area covers 17500 hectares and contains three freshwater lakes, Kanyaboli, Sare, and Namboyo. Part of Yala swamp covering about 2300 hectares has been reclaimed for rice production by the Dominion group of companies. The total annual rainfall in this region averages 1400 mm with the first peak between March and April and second peak between November and December [8]. Most of the inhabitants of Kanyaboli Village live in traditional houses with mud walls and grass thatched roofs. The eaves of most houses are open allowing for unimpeded entry and exit of mosquitoes, which bite the unprotected humans sleeping in these houses. Family compounds, consisting of one or more houses are separated from each other by farmland. Apart from working in the rice fields under contract at the Dominion farms, the inhabitants also practice subsistence agriculture, growing crops such as maize, millet, and cassava. Fishing is carried out on small scale in the lake to be eaten as a source of protein and sold to supplement for monthly family income. The Domion farm has cooperated with malaria programs to gradually cover the villages around with bed nets over a time frame of 3 years at the expense of the farm. In cooperation with the net producer, we offered our bed nets at cost price to two steps of this campaign, for the intervention village at start and for the control village 6 months later. CDC in Kisumu had previously been interested in this area and found that the malaria vectors in the area are *Anopheles arabiensis* and *funestus* with a year round transmission recorded at a local clinic (O. Skovmand, pers. comm.). The Dominion farm had established the clinic in a village near the farm building complex and paid the staff and other costs. It receives patients from all the villages around the farm area and the farm staff. Malaria was identified on clinical manifestations and treated according to national guidelines. Our co-operation with the clinic thus allowed for passive detection of malaria cases. 

### 2.2. Study Design

A sample size of 150 houses in 150 compounds matched in size was selected through simple random sampling by lottery at the intervention village. Long lasting treated mosquito bed nets Netprotect were hung in the selected houses in December 2007. Two–Five LLINs were put up by the study group per household for full coverage of all sleeping places. A compound consisted often of a main house and one or more small cottages for teenagers or elder people. Typically, there would be two sleeping places in the main house and one or more in each of the cottages. Only the nets in the main house were followed after the first month. To cater for mosquito flight range, another 150 houses were randomly selected 2 km away in the adjacent area to act as control arm where untreated bed nets were distributed. After 6 months, the households of the control area received around 450 LLINs of the same type. The trial thus fit with the local plan for a gradual expansion of bed net coverage in the villages around the swamp.

### 2.3. Sampling of Houses for Mosquitoes and Species Identification

Houses for sampling were selected by two-stage cluster sampling each month upon the consent of their owners. The first stage was the selection of a cluster of houses within the village. The second stage was random selection of one house and the nearest ten neighboring houses with probability proportional to size within each cluster in the village were then included in the sample. Indoor resting adult mosquitoes were monitored with a pyrethrum spray catch (PSC) between 7:00 am and 10:00 am. White sheets were spread on the floor and the furniture within the houses. Two collectors, one inside the house and one outside, sprayed the selected houses with 0.025% pyrethrum with 0.1% piperonyl butoxide emulsified concentrate. The collector outside sprayed around the eaves, while the one inside the house sprayed the roof and the walls. The house was then closed for 10 minutes, after which knocked-down mosquitoes were collected from the white sheets indoor and put on moist filter paper in Petri dishes. Each Petri dish was labeled as per house identification number and packed in a cool box. The collected mosquitoes were transferred to the laboratory at Kenya Medical Research Institute (KEMRI) in Kisumu. All *Anopheles* mosquitoes were identified to species level using morphological keys [9]. *An. gambiae s.l.* and *An. funestus* were then sorted out based on the abdominal status and characterized as fed, unfed, gravid, or half gravid then stored in vials containing anhydrous calcium sulphate. A record sheet was completed detailing gonotrophic stages, sex, and species identification. 

### 2.4. Collection and Preservation of Blood Fed Mosquitoes

All blood fed mosquitoes from each collection were preserved in labeled vials containing anhydrous calcium sulphate. Samples of blood fed mosquitoes were cut transversely between the thorax and the abdomen for all posterior portions containing the blood meal. The abdomen of each mosquito was ground in 50 *μ*L of phosphate-buffered saline (PBS) pH 7.2 with subsequent addition of 950 *μ*L of PBS and then stored at −20°C in the refrigerator.

### 2.5. Mosquito Blood Meal Analysis

Blood meals were identified by a direct enzyme-linked immunosorbent assay (ELISA) at KEMRI in Kisumu, using anti-host IgG conjugates (Kirkegaard and Perry, Gaithersburg, MD) against human, bovine, chicken, and goat according to a protocol by Beier et al. [10]. 397 blood fed *Anopheles* mosquitoes were tested by ELISA technique for the origin of their blood meal. 

### 2.6. Polymerized Chain Reaction for the Identification of **Anopheles gambiae ** Complex

Individual mosquito specimens from field collections were prepared for identification by removing a leg with sterile forceps and placing it into one well of a 96-well PCR tray. Each well contained 100 *μ*L of grinding buffer (0.10 m NaCl, 0.20 m sucrose, 0.30 m Trizma base 0.01 m EDTA, and 100 mL sterile water pH 8.0). Trays were covered securely with sterile adhesive foil and placed in water in a sonicator bath (Bransonic ultrasonic cleaner, Shelton, CT, USA) at 65°C for 20–40 minutes. To these trays 14 *μ*L of 8 M potassium acetate was added, and the mixture was placed in cool ice to precipitate the protein. The mixtures were microfuged, top speed, for 30 minutes, and the resulting supernatants were transferred to new tubes. 200 *μ*L cold 95% ethanol was added to samples and placed in a freezer for at least 20 minutes to precipitate the DNA. Samples were washed with 200 *μ*L 70% ETOH followed by 200 *μ*L 95% ETOH, and tubes inverted for complete drying (about 1 hour). 1 *μ*L DNA samples and controls for *An. gambiae* or *An. arabiensis* were added to wells. Blanks contained master mix in ice only. 14 *μ*L master mix (Water, taq enzyme specific mosquito primers Mgcl_2_, and 10x buffer) was added to all wells followed by 4 drops of heavy mineral oil to overlay the reaction mixture. The plates were then covered with a plate sealer and placed in gene machine. All conventional PCR reactions were performed using the Epicentre FailSafe PCR System. The reaction program had an initial step of 80°C for 1 min, followed by 30 cycles of denaturation at 94°C for 30 seconds, annealing at 50°C for 30 seconds, and extension at 72°C for 30 seconds, with a final extension at 72°C for 4 min. The PCR products were separated by electrophoresis on 2% agarose TBE gels and stained with ethidium bromide. The amplicons were visualized with an ultraviolet transillumination gel documentation system.

### 2.7. Determination of Malaria Incidens

Malaria cases were detected passively at the village clinic established by the nearby Dominion farm. The two villages of our study were about 2 km away from the clinic. People seeking treatment were compared to the list of people participating in the study either in intervention group or control group but with status blind to the clinic personnel. No other records of malaria cases were used, and self-treatment in households was not followed. Malaria was identified using clinical manifestation, and we added rapid diagnostic testing after training the staff. Rapid diagnostic tests (RDT: Paracheck PF tests kits) were carried out according to the manufacturer's instructions. Children under five years who were clinically identified as malaria positive were treated, irrespective of the results for Paracheck test in accordance with the national guidelines. Children older than five years and adults were first examined and scored clinically then tested with Paracheck but only treated according to MOH guidelines in 2007 amodiaquine (10 mg/day) for 3 days if a Paracheck result was positive for malaria [11]. These guidelines were changed in 2010, and the study is thus not concerned. Malaria incidence rate per month was determined.

### 2.8. Counting, Testing, and Sampling Nets in the Field

The field counting of nets took place 4 days, one month, and 35 months after nets hanging. Net hanging above a bed or told to be used were counted as nets in use. For the months from 1 to 9, bioassays were made on nets in the field by placing cones with lab reared, transported mosquitoes on the nets as they were hanging [12]. The collections of nets after years 1, 2, and 3 actually took place after 18, 28, and 38 months, where 34–38 nets were collected and brought back to the laboratory for further evaluation. All the nets brought back to the laboratory were examined for damages (see below). Removed nets were replaced with new nets, same size but in a different color. For some or all of these, 5 swatches of 30 × 30 cm were cut according to WHO guidelines (from the four sides and the roof) and used for biological and/or chemical assays. Because bioassay capacity in the laboratory in Kisumu showed to be limited, the 18-month samples were sent to a larger bioassay laboratory in Chiang Mai, Thailand. Samples collected after two years were sent for chemical analysis only. Among the 30 nets collected after 3 years, 10 bed nets were randomly sampled square pieces measuring 30 × 30 cm were cut from the sides and tested in bioassays in Kisumu. Chemical analyses were made on 2 of the 5 samples of the nets collected this year. The rest of the samples were stored at room temperature for 3 years and analyzed again in the same laboratory. In 2009, IIC established in-house chemical analysis facilities in Hanoi, Vietnam, that were used for the 3-year samples. Samples analyzed before that were performed by Hanoi University. Details of protocols are given below.

### 2.9. Determination of Residual Insecticidal Activity of Netprotect

Standard WHO cone bioassays were performed on random samples of Netprotect monthly from month 3 to 9 after installation using a laboratory colony of susceptible strain of *An. gambiae s.s. kisumu* strain, 3 days old females. A bioassay cone was attached to one side of hanging bed nets in the houses. One hundred mosquitoes in replicates of ten were exposed to each bed net in ten randomly chosen houses using standardized procedures. After 3 minutes of exposure, mosquitoes were transferred to 200 mL labeled paper cups, provided with 10% sucrose solution, brought back to the laboratory, and maintained at 28°C ±2, RH 80% ± 10%. Knocked down at 60 minutes, KD60 were observed in the field and percentage mortalities after 24 hours were recorded in the lab. A similar procedure was also performed for untreated nets as a control sample. The corrected mortality was determined using Abbot's formulae. 

The nets swatches from 18 months were sent for bioassay at the University of Chiang Mai that made standard WHO cone test with 3 min exposure on 2 of these swatches per net, randomly chosen among nets, but with known position. Readings were 1 hr knock down and 24 hr absolute mortality. 50 females, 2–4 days old *Anopheles cracens* (former *dirus B*), were used for these bioassays per sample, procedure as described above. 

10 to 14 *Anopheles gambiae s.s. kisumu* female mosquitoes were exposed to net swatches from year 3, following the procedure described above; the lower number due to the limited test capacity in Kisumu. Two criteria for mortality were applied, absolute mortality where the mosquito does not move when touched, and functional morality, where it has lost at least 3 legs or one wing, and therefore will fall to the ground in a house and be eaten by ants.

### 2.10. Chemical Analysis

The nets were delivered by the producer Bestnet A/S with a chemical analysis made by the Singapore Laboratory also used by Crown Agent and UNICEF. In our analyses, the net swatches analyzed per net were cut to pieces, and a subsample was drawn for extraction in xylen under reflux following the WHO protocol for deltamethrin incorporated PE nets. Hanoi University used the HPLC method according to CIPAC 333/LN [13], whereas the IIC laboratory used GC FID to analyze extracts of the net as recommended by the CIPAC/WHO reference laboratory in Gembloux, ISO 17025. This method is mostly the same as the CIPAC method except that for the GC FID the xylen is not evaporated followed by a dissolve of the insecticide in injection solvent; the xylen solution is injected directly. Procedures for this method are very close to these of GC-*μ*ECD recently published by the same laboratory [14]. 

### 2.11. Net Damages and Use Parameters

4 days after the initial distribution, 1 month and 36 months later, all households in the first distribution area were revisited to see if the nets were still hanging. If not hanging, the owners were kindly asked where the nets were and if they were still in use. Responses were tabled according to category. After years 1, 2, and 3, around 60 households were visited, and net presence and state (holes, dirtiness) were recorded. 34 to 38 of these nets were randomly picked (by tossing a coin before entering the house) from the first village and brought back to the laboratory. However, it was found that especially smaller holes were underestimated in the field and only data from the nets collected and brought back to the laboratory are presented below. For each of these houses, the following information was taken: the net user, number of rooms in the house, whether the kitchen was in the same room as the net taken (fire damage), and how often the net was reported to be washed. The nets were suspended on a tube frame borrowed from CDC in Kisumu, and holes were counted and sizes estimated in 5 categories: below 1 cm, 1-2 cm, 3–5 cm, 5–10 cm, and above 10 cm. These categories were chosen before WHO started working with hole indexes. Holes were divided into tear hole and burn holes; the latter is recognized from the borders of the holes. This scale is different from the one recently introduced guidelines from WHO on long lasting insecticidal nets [15] since our data were collected before this scale was published. In the WHO hole index, holes below 0.5 cm are discarded, category N1 holes are 0.5–2 cm, category N2 are 2–10 cm, N3 are 10–25 cm, and N4 are holes above 25 cm. The number of holes per category is multiplied with an index factor (1, 23, 196, and 576, resp.), and the sum is divided by the numbers of nets. Since we had “above 10 cm” as the largest group, we arbitrarily divided these holes into two groups, (1/2) 10–25 cm and (1/2) above 25 cm. The WHO index is used for relative comparison between products tested and provides no categories for the values found. 

The net height was measured in the 4 corners of the net suspended on the frame and averaged per net.

### 2.12. Data Management and Analysis

Comparison of proportions between categorical variables was performed by chi-square test at 95% confidence level using SAS statistical software version 9 [16]. Repeated Poisson regression using the GENMOD procedure in SAS was used to analyze the effects of the bed nets on human feeding success on blood meal for the *Anopheles* mosquitoes found resting indoors. For each model, the number of indoor resting mosquitoes in control houses was the reference. The percentage reduction was calculated as one minus the relative risk as estimated by Poisson regression. Odds ratio was also used to determine the difference between densities of the *Anopheles* mosquito species collected in treatment and control villages. General linear variance analysis was used to estimate parameters influencing efficacy measured in bioassays after 2 years using Statistix, version 9 [6]. Linear regression analysis was used to estimate the impact of reported number washes on deltamethrin loss, epimerization, and net height. 

### 2.13. Ethical Clearance

The Netprotect project was reviewed and approved by KEMRI and Kenyatta University Graduate School Board. Informed consent was sought from the individuals who participated in the study. This was done after the explanation of objectives and collection methods through individual discussion and group meetings.

## 3. Results

### 3.1. Density of **Anopheles ** Mosquitoes Resting Indoor

A total of 807 indoor resting female adult *Anopheles* mosquitoes were collected from 80 randomly sampled houses during the first 6 months of study. Out of these, 82.5% of the mosquitoes collected were from the control houses while 17.5% from the intervention ones. Two species of *Anopheline* mosquitoes were identified from the specimens collected, *An. funestus* as the most abundant species (69.9%) and *An. gambiae s.l.* (30.1%) (Table 1). 

Polymerized chain reaction (PCR) identification of members of *An. gambiae* species complex revealed that all the 243 *An. gambiae s.l.* analyzed were *An. arabiensis*. There was a significant difference in numbers of* An. arabiensis* found in the control houses compared to the intervention houses (*χ* ^2^ = 22.6, df = 1, and *P* < 0.001). The probability of finding *An. arabiensis *in the control area was 2.6 times higher than that in intervention area (OR = 2.6, CI: 1.9–3.9). A total of 564 *An. funestus* were collected; 13.3% of them were from the intervention houses and 86.7% from the control houses. There was a significant difference in densities of *An. funestus* between the two areas (*χ* ^2^ = 22.63, df = 1, and *P* < 0.0001), and there were 6.5 more chances to catch an *An. funestus* in a compound in the control area than in the intervention area. 

### 3.2. Indoor Resting Patterns for Blood Fed **Anopheles ** Mosquitoes at Kanyaboli

There was a tendency of more indoor resting blood fed *An. arabiensis* (*χ* ^2^ = 2.99, df = 1, *P* = 0.084) collected from control than from intervention areas (Table 2). Densities of indoor resting engorged *An. funestus* were significantly higher in control area compared to the intervention area (*χ* ^2^ = 17.44, df = 1, and *P* < 0.0001). The likelihood of finding a blood fed *An. funestus* in the control area was four times more than that of finding a fed *An. funestus* in the intervention area (OR = 4: 95%; CI: 2.0–7.2). 

### 3.3. Blood Meal Origin of Indoor Resting **Anopheles ** Mosquitoes

A total of 515 blood fed *Anopheles* mosquitoes were tested by ELISA technique for the origin of their blood meal. 65% had their blood meals identified, 258 *An. funestus* and 139 *An. arabiensis *(Table 3). In the intervention area, 53% of the *An. funestus* were positive for bovine antibodies, 29% for human antibodies (IgG), and 2% had mixed blood meals human/bovine. In the control area, 46% of *An. funestus* were engorged on human blood meal, 29% were positive for bovine blood meal, and 2.2% engorged on mixed blood meals of either chicken/human or bovine/human, and 23%, blood meals could not be identified with our methods. The change in blood meal origin for *An. funestus* is highly significant (*χ* ^2^ = 30.7, df = 1, and *P* < 0.001). 77% of *An. arabiensis* sampled from houses with LLINs fed on bovine host, to a lesser extent on mixed human/bovine (5.7%), and none (0%) was positive for human blood meal. In the control villages, 42.3% of the *An. arabiensis* were positive for bovine blood meal and 4% for human blood meal, 7.7% had mixed blood meal of human/bovine, and 1% had mixed blood meal of bovine/chicken (Table 3). This difference is not significant.

### 3.4. Residual Insecticidal Activity of Netprotect

Bioassay results on 10 randomly sampled bed nets showed a knockdown rate and absolute mortality rate of 80% and 100% respectively, up till seven months of use. However, after 9 months of use, 2 sampled nets showed 24 hr mortality below 80%. Around 50% of the mosquitoes exposed to these 2 bed nets were still alive 24 hours later but had lost three or four of their legs, respectively, thus were “functionally dead.” 

After 18 months, 38 nets were brought to the laboratory for damage estimates (hole counts), and from 22 of these, 2 among 5 swatches were analyzed in bioassays. Using a threshold of 50% mortality as done by Lindblade et al. [12], 13 nets passed in both samples, 6 passed in one and not the other, and 3 failed in both. Two of these 3 were very dirty; see below. General linear variance analysis showed that net code was a significant parameter for knockdown for 1 hr (df = 21, *F* = 3.2, and *P* = 0.008), whereas swatch position was not significant (df = 4, *P* = 0.85). For 24 hr mortality, net code was significant (*P* = 0.006), whereas position was not significant. Mean KD1Hr was 86%, median was 96%, and mean mortality was 68% with median 67%. Three nets (6 swatches) were very dirty when tested and gave lowest mortality recorded, 10 to 40%. The general AOV analysis showed that “very dirty” was a highly significant parameter (*P* < 0,001) for bioefficacy. These swatches were washed and tested again after 3 days. For 2 of these, this reduced the efficacy to near 0, but for the third, it increased from 10% to 96% mortality. If these very dirty nets were removed from the mean values, KD1Hr became 91% and Mort 24 hr 76%. 

After 3 years, bioassays were performed on 10 nets randomly picked from the 34 nets taken to the laboratory and tested for bio-efficacy. Eight of them provided 100% killing efficacy, one 85%, and one 76%; however, KD1Hr was 100% for these two nets, so the 10 nets passed.

### 3.5. Chemical Data

Chemical data are summarized in Table 4. Start concentration of deltamethrin was determined by the provider, Bestnet A/S, before the nets were sent to Kenya and determined to be 1.90 g deltamethrin/kg net.

5 net samples taken after 9 months were sent for chemical analysis after the bioassay. Three of these induced mortality samples were between 95, and 100% and two below 80%. The three with high mortality contained 0.999, 1.191, and 1.529 g deltamethrin/kg, whereas the two that gave low mortality had 1.164 and 0.976 g/kg, thus not clearly lower.

Twenty-four and 34 nets were sampled for chemical analysis at the end of year, 2 and 3, respectively. These nets were among the nets taken for hole analysis, so a description of each net (general state including loss of color, holes, and dirt) and the chemical analysis are known as the comments of the owners on the nets, including informed date of last washing. Average deltamethrin was 0.77 g/kg plus 0.30 g R-isomer/kg net. Among the 34 nets taken after 3 years of use, 3 nets had no insecticide content. One of these had no color at all indicating the use as fishing net or outdoor use for long time. One looked as brand new (shiny blue), had no label, and was probably not a Netprotect. The third one looked normal. Thus, the two of these three are not included in the calculations. The data show that deltamethrin content had declined to mean 0.73 g deltamethrin/kg net plus 26% isomer after 2 years use and 0.49 g deltamethrin/kg net with 49% isomer after 3 years (Table 4).

One net with a value of 0.11 g deltamethrin/kg net had the comment of the owner that then mosquitoes were biting him the fact despite that he used the net.

To estimate the impact of net storage after sampling, net swatches from 15 nets collected in year 3 were analyzed 3 years later. A pairwise analysis (analyses of swatches from same nets paired) showed that storage at room temperature for 3 years did not provide a significant decline in insecticide content. The mean content after 3 years of use and 3 years in storage was 0.33 g deltamethrin per kg net compared to 0.44 g from the same nets determined shortly after collection.

The frequency of net washing was reported from two questions: how often this net is washed and when this net was washed last time. From the responses, the number of washes over 3 years was estimated per net. Among the 34 nets taken to the laboratory for closer examination, 3 came from houses where the response was “do not know,” therefore, chemical data from these houses are omitted in the correlation study to wash frequency. Reported frequency of washes at the end of the third year was contrasted to chemical content and net height, two parameters that might be expected to change in a polyethylene net with the number of washes. Net-height data are reported below. Number of washes varied from 36 to 0 over the 3 years, with a mean of 5.8 and median 3, meaning once per year. There was no correlation between deltamethrin content and number of washes (*P* = 0.22). However, when data for R-isomer content was correlated to number of washes, the correlation was highly significant (*P* = 0.009), though the correlation coefficient was moderate (*r* ^2^ = 0.51).

### 3.6. Net Damages: Holes

Nets taken back from the field were inspected for holes when hanging on a frame. The size of holes and their position from the border were noted as summarized in Table 5. Around 50% of holes were below 1 cm and were caused by burst of a few yarns or by sparkles. Including all hole sizes, the number of holes per net varied from 5.48 in the first year, to 4.09 in the second year, and 11.06 in the third year. About equal numbers were burn holes and tear holes. The number of holes on the lower 30 cm increased from 45% in the first year to around 70% in the second and third year. Mean hole index as defined according to the new WHO bed net hole index was 333, 114 and 381 for the 3 years, respectively, but the standard deviations are much larger than the means. Median index values were 24, 3, and 61 for the three years. The number of nets with no holes present was nearly constant around 15% (year 1: 6/38, year 2: 6/34, year 3: 5/33, excluding the new looking, hole free, and probably not Netprotect net).

We examined if burn holes were determined by the number of rooms of the house, based on the hypothesis that, in a one-room house, the net would be together with the kitchen fire. However, this association was not significant (*P* = 0.30). Therefore, in the year 3 it was tabulated if the kitchen fire was in the same room as the net taken back to the laboratory for holes examination. Again, this analysis did not reveal the impact of this coexistence on any burn hole found. Finally, using the WHO index for burn holes only and pairing this index per net with the presence of kitchen fire in the same room were not significant either (*P* = 0.16).

### 3.7. Net Use

All households were revisited 4 days after the net hanging campaign and again one month later. After 4 days, the houses of 398 nets were found; one house with two nets could not be found. Twenty nets could not be inspected because the owners were absent. Among the rest (378 net), 22 nets were missing.  Table 6 gives the responses for not hanging. The most common reason was that nets were given away or sold. The second most common reason was that the roof leaked where we had hung the net, so it was taken down. 

After 1 month, 73% of all nets were seen hanging, but we had no access to observe 37 nets because the owners were absent. Among 35 nets missing, 21 had been given away or sold, and 5 had moved with the net user; 4 of these were kids that had gone to a boarding school. Three nets had been discarded because of fire damage. 

After 35 months, the study population was 398 minus the 35 missing after one month. We had removed 38 nets for inspection after 18 months and 34 after 26 months. The remaining nets were thus 291 at the last counting in November 2009. Among these, a further 35 were missing. Nine had moved with user, 4 with kids going to boarding schools, and 26 were not traceable, either discarded or used for other purposes. Attrition rate including nets moved with users was thus (35 + 35)/(398 – 38 − 34) or 21 %, excluding nets moved with users, 16%.

Net height was correlated to reported washings. The average net height after 3 years of use was 140 cm (nets purchased for the study were 150 cm) with a variation from 120 cm (one net) to 150 cm, but there was no correlation to the number of washes.

### 3.8. Impact of Netprotect on Malaria Incidence

**Figure 2:**
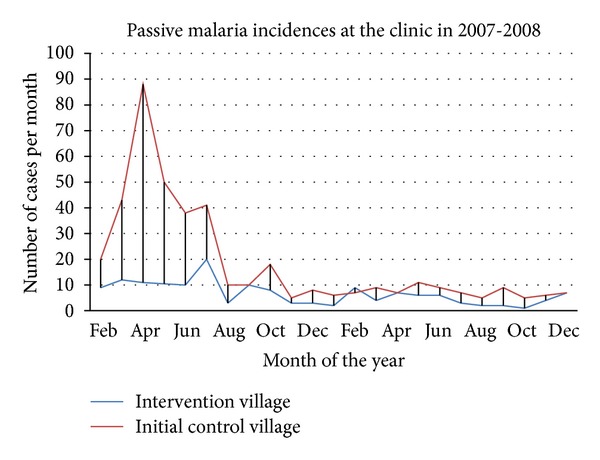
Monthly malaria cases in Kanyaboli area before and after intervention. *Month of Netprotect hunging in control area.

 The total number of people living in the compounds was counted as a part of the study preparation. 1350 living in compounds were used for the study in the intervention area, and 1622 in the compounds in the control area of included in this study, total 2972. 1234 inhabitants living in compounds of the study or the control areas came to the clinic and had the diagnosis, malaria based on clinical manifestations between January and July in 2007. From the intervention area, 220 had malaria diagnosis, and 67 of these were confirmed by rapid test kit (RDT, Paracheck). From the control area, 670 patients had diagnosis of malaria; 277 of these were confirmed by RDT. Malaria incidence among people seeking the clinic thus was 41.3% in the control area and 16.3% in the intervention area during this period. The overall effect of Netprotect on malaria prevalence was significant between the two areas before the bed nets were also distributed in the control area (*χ* ^2^ = 8.28, *P* = 0.004). The peak outbreak of malaria cases in the control area was recorded in April in the larger rainy season with a total 88 cases (Figure 2). However, malaria cases in intervention area remained stable at a low level with a small peak in July. Netprotect bed nets were then distributed to the control area at end of July 2007 and malaria incidences followed passively at the local clinic for 2 years. The peak transmission could no longer be seen the second year in any of the areas. In the third year, the clinic became involved in the distribution of nets to pregnant women, and incidence could no longer be attributed to Netprotect only. 

When comparing malaria identification from clinical manifestations and from rapid test kits (Paracheck), fewer were confirmed by the RDT in both areas, and the ratio (confirmed by RDT/confirmed by clinic) was the same for the two areas and constant over time. For the first 6 months, clinical manifestation revealed 3.1 more cases in the control area than in the intervention area, RDT 3.7 times more. These differences between intervention and control areas are significant for both methods (*P* < 0.05). Further, overestimation of malaria incidence from clinical manifestations was not influenced by incidence level. 

## 4. Discussion

The results showed that Netprotect had a significant impact on densities of indoor resting *An. arabiensis* and *An. funestus* in Kanyaboli village. The number of indoor resting *Anopheles* mosquitoes was significantly lower in the intervention houses compared to control houses (Table 1). The probability of finding *An. funestus* in the control area was 6.5-fold higher than that in the intervention area, and the probability of collecting *An. arabiensis* in the intervention area, however, was 2.6 times greater compared to that of the control area. This conforms the results reported in Gambia, Sierra Leone, and Kenyan coast studies on ITNs and curtains which demonstrated decreased indoor resting densities when pyrethrum spray catch (PSC) was used as the collection method for *Anopheles* mosquitoes [1]. However, while ITNs exhibit rapid loss of efficacy unless retreatment is adhered to every six months, this study shows that Netprotect efficacy on *Anopheles* mosquito was high without the necessity for re-treatment during the project period. 

*An. funestus s.l.* was found to be the predominant vector resting indoors in this area constituting approximately 70% of the total mosquitoes collected indoors;* An. arabiensis* made up the remaining 30%. *An. funestus* was strongly affected by the net campaign (reduction of 65.5%, *P* < 0.001) compared to *An. arabiensis*. Similarly, studies conducted by Gillies and Coetzee [17] demonstrated that this species was highly susceptible to chemical control measures and was slow in recolonizing an area from which it has been controlled. 

There was increased degree of zoophily in houses where Netprotect was in use (Table 3). *An. arabiensis* is known to be zoophilic but also bite humans and rest indoor, whereas *An. funestus* is known to be anthropophilic and rest indoor. Introduction of treated bed nets significantly changed blood feeding of *An. funestus* but not of *An. arabiensis*. *An. funestus* is considered very anthropophilic, so this effect is a warning about its flexibility at high bed net coverage.

Alternatively, a fraction of the *An. funestus* population feeds on cattle and is not much influenced by the use of bed nets. *An. funestus* is a complex species, but it was not tested if the bovine feeding was different from that feeding on humans.

Three minutes exposure of bioassays on Netprotect showed a mortality of 100% during the first seven months of net use and the failure of a few. Chemical analysis of the nets that failed to those that did not did not reveal a dose difference. Further, bioassays on two samples per nets with known positions and taken from 30 nets did not reveal an effect of net position, not even from the roof. The general idea that the roof is less exposed to handling and that handling is a major reason for insecticide loss can therefore not be confirmed from this study.

Results after three years of ten randomly sampled bed nets indicated that they were still highly efficacious. Ten out of 10 nets gave a KD1Hr of 100%. 

Bioassay data from the three years were not made with the same *Anopheles* species, and data produced using *An. gambiae* strain *kisumu* gave higher mortality than those obtained with *An cracens* independent of net age. When running the test for insecticide resistance, WHO use a different thresholds for different species of *Anopheles*; however, when running these 3 min exposures, the known difference in sensitivity is not considered. For comparison, we sent a net to the WHO reference laboratory LIN in Montpellier in 2012 that gave 60% mortality with *An cracens,* and this gave 100% mortality at LIN with *An gambiae* strain *kisumu* (data not shown). Since the LIN is the reference laboratory for these evaluations, the data generated with *An gambiae* strain *kisumu* are the most relevant. However, the large amount of bioassays with *An cracens* relating effect to the position on the net is still valid information.

Loss of efficacy on other LLINs has also been cited to be due to external factors such as dirt and fume accumulation on the net fabric [18]. Three of 24 nets tested after 9 months were extremely dirty and showed low mortality. They were washed and tested after 3 days, and still two failed, but one jumped to 96% from 10% mortality. Impact of dirt is thus not always the same, neither the effect of washing dirty nets. 

Chemical analysis of 30 nets after 2 and 3 years use revealed a decline in deltamethrin from the original 1.9 g deltamethrin to 0.49 g/kg net after 3 years (Table 4). Loss in deltamethrin was not depending on the number of washes reported but deltamethrin R-isomer content was. The reason might be that the main reason for loss of deltamethrin is evaporation, but the washing process or the drying in the sun after washing causes the epimerization. UV light is known to cause deltamethrin epimerization. Five series of nets from a test program of test recipes showed that the amount of R-isomer was constant measured after 0, 5, 10, 15, and 20 washes (O. Skovmand, prs communication). Washing is therefore not considered as causing epimerization of deltamethrin in a polyethylene matrix. The reported number of washings is not off course a very solid parameter, but the significant correlation to the degree of epimerization indicates that it has value.

A single complaint of net failure from a user showed the net content to be 0.11 g deltamethrin/kg net. More data are needed to know if this represents the whereabouts of a threshold for field failure.

Bed net use rate was measured for all 400 nets in the first intervention village after 4 days, one month (Table 6), and 3 years. The major reason for not seeing a net hanging was that we could not enter the house where it was supposed to be. If the use rate among these people is estimated to be the same as that in these houses, then 95% of all nets were hanging after 4 days and 90% after a month. The second most common reason for not finding a net was that it was given away or sold, and the third (after 1 month) was that the net had moved with the owner. If these latter nets are supposed to be in use, the use rate after one month is 91%. Net gone was explained as the net being sold to generate supplementary income or donated to relatives from other regions. 

After 35 months, further 35 nets were gone, 9 moved with the owner and 26 were missing, either discarded or used for other purposes. Again anticipating that the 14 nets moved with owners were in use with the same frequency as those remaining, around 87% of the nets were still in use after 3 years, which is a high percentage. It should be mentioned that this collection was in November therefore in the smaller rainy season. 

The number of holes did not increase significantly between years one and two (Table 5) but did increase dramatically after three years of use. We examined if burn holes could be correlated to the presence of kitchen fire in the same room, but such a correlation was not found. The use of small open fire oil can lamps is thus probably a better explanation. Tear holes were mostly on the lower 30 cm are that mostly tucked under the mattress. Tear holes from the lower 30 centimeters increased dramatically the third year as an indication of daily use. Only a few nets were repaired.

The hole index was calculated per net and mean; standard deviation and median were calculated according to WHO guidelines [15] (Table 5). However, these values were not normal distributed and the standard deviation per year was always much bigger than the mean. Bigger holes have a multiplication factor of 576, so a single hole of that size in one net has a dominant effect on the mean. Neither of the median values seems to reflect an evolution since it went from 24 to 3 to 61 for the 3 years. Contrary to this complex model, simply counting the number of holes showed an increase from year 1 to year 3. That number of holes increased, and the WHO hole index did not show that nets with big holes were discarded preferentially, which off course make sense. But it may also show that the new WHO hole index is too sensitive to big holes and shows random variation in values caused by these rarely found nets. 

This study was planned as a three-year study. However, in the third year, an antenatal program of distributing another polyethylene net started, and the mass effect could no longer be ascribed to the polyethylene net of this study. Therefore, we only present reported malaria cases from the first 2 years.

The study collected morbidity results on passive and clinic-based cases and showed a significant difference in malaria cases during the six months of trials between the original intervention area and the original control area. The incidences rate was higher in the control village (41.3%) compared to intervention area (16.3%). When bed nets were used in both areas, the incidences of malaria cases in the two areas were no longer different. In the second year, malaria incidence as measured here remained low in both areas (Figure 2). Of interest, the peaks of malaria incidence nearly disappear and what remains is year round “background” malaria not influenced by the bed nets.

This study has a weakness. Preintervention data were not collected from the two parts of the village where the study was carried out. However, during the first 6 months of the trial, no LLINs were distributed to the control area, and 450 nets were distributed after these 6 months. We thus have pre-intervention data from the control area. When LLINs were also distributed in the control area after 6 months, the mosquito density and malaria prevalence mirrored these of the of intervention area (Figure 2). Further, the villages were in the same environment around a swamp with no observed differences in breeding sites and with approximately the same distance of 2 km from the clinic.

## 5. Conclusions

This study demonstrated lower indoor resting density for both *An. arabiensis* and *An. funestus* mosquitoes in the intervention area. There was lower human blood feeding index in intervention area compared to the control area. Bed nets were still very effective in killing mosquitoes after three years of use. Malaria prevalence in the intervention area was at a low, stable level despite seasonal malaria variation observed in the control area. The disappearance of the seasonal peak was observed in both the areas the following year where the LLINs covered both areas. After 3 years, 84% of nets were still found hanging or being washed, and a further 3.5% had moved with the owner. Bioassays with *An. gambiae kisumu* strains showed nets to be effective with KD1Hr at 100% mortality after 3 years, use, but a large series of bioassays with *An cracens* after year 1 showed that 1 net out of 19 nets failed in bioassays from both of two samples collected per net on a 50% mortality acceptance criteria. *An. gambiae* strain *kisumu*, however, is the fully sustainable strain used for WHOPES evaluation of long lasting nets. Chemical analysis showed a decline from 1.9 g to 0.49 g deltamethrin/kg over 3 years of use.

## Figures and Tables

**Table 1:** Indoor resting patterns of *Anopheles* mosquitoes by species at Kanyaboli.

Mosquito species	No. of mosquitoes sampled in		Reduction %	Totals (%)
	Control area (%)	Intervention area (%)		
*An. arabiensis *	177 (72.8)	66 (27.2)	62.7	243 (30.1)
*An. funestus *	489 (86.7)	*75 (13.3) *	84.7	564 (69.9)

Totals	666 (82.5)	141 (17.5)	78.9	807 (100)

Percentages are in brackets.

**Table 2:** Indoor resting patterns for blood fed *Anopheles* mosquitoes.

Mosquito species	Control area	Intervention area	*P* value	*χ* ^2^-value	Odds ratio
*An. arabiensis *	66	39	0.084	22.98	—
*An. funestus *	308	41	<0.0001	17.4355	4 (2.0–7.2)

**Table 3:** Blood meal origin of indoor collected* Anopheles* mosquitoes.

Mosquito species	Sampling area	No. tested (*n*)	% of mosquitoes positive for vertebrate blood meals					
			Human	Bovine	Goat	Chicken and human	Human/bovine	Chicken/bovine
*An. funestus *	Intervention	34	29	53	0	0	0.2	0
*An. arabiensis *	Intervention	35	0	77	0	0	5.7	0
*An. funestus *	Control	224	46	12.5	0	2.2	2.2	0
*An. arabiensis *	Control	104	3.8	42.3	0	0	7.7	0.9

**Table 4:** Chemical analysis of bed nets collected after 2 and 3 years of use.

After months of use	No. of samples analyzed	Deltamethrin ± Std Dev (g/kg)	R-isomer (g/kg)	Median deltamethrin (g/kg)
0	4	1.90 ± 0.02		
9	5*	1.17 ± 0.22	0.21 ± 0.02	1.16
24	22	0.73 ± 0.53	0.30 ± 0.22	0.74
36	31	0.49 ± 0.48	0.48 ± 0.23	0.36

The 5 samples represented 3 with high bioassay efficacy and 2 with low.

**Table 5:** Net damages and WHO hole index after 3 years of use.

Netprotect	1.5-year use	2-year use	3-year use
Number nets	38	34	34
Nets with no holes	6	6	5
Nets with burnt holes	18	16	27
Nets with tear holes	17	19	25
Total number of holes	115	99	409
Burn holes/all nets	3,03	2,13	2,74
Tear holes/all nets	2,45	1,96	8,32
Pct holes lower 30 cm	48%	73%	70%
Pct holes < 3 cm	70%	77%	72%
Pct holes < 1 cm	53%	53%	43%
Average WHO hole index	333	114	381
Std Dev	634	188	656
Median	24	3	61

**Table 6:** Reasons for nets not found hanging.

Reponses	4 days after distribution		1 month after distribution	
	Numbers	Percentage (%)	Numbers	Percentage (%)
Owners absent	20	48	37	52
Netprotect given away/sold	7	17	21	29
Leaking roof, net taken down	4	10	0	0
Use old net	3	7	4	6
Net too short	3	7	0	0
Too smoky in kitchen	2	5	0	0
Net provokes “Allergy”	1	2	0	0
No bed	1	2	1	1
User gone with net	1	2	6	8
Too damaged due to fire	0	0	3	4

Total	42	100	72	100

The main reason for nets not found was that the owner was absent. The second reason was that nets were given away or sold. After one month, school had started and 5 kids had gone to boarding school with their nets.

## Abbreviations

CDC: Centre for Disease Control
ITNs: Insecticide treated bed nets
KEMRI: Kenya Medical Research Institute
KNBS: Kenya National Bureau of Statistics
KWF: Kenya Wastelands Forum
LLINs: Long lasting insecticide treated bed nets
MoH: Ministry of Health
PSC: Pyrethrum spray catch
RDT: Rapid diagnostic test
SAS: Statistical Package for Social Sciences
WHO: World Health Organization
WHOPES: World Health Organization Pesticide Evaluation Scheme.
